# Edible and Herbal Plants for the Prevention and Management of COVID-19

**DOI:** 10.3389/fphar.2021.656103

**Published:** 2021-04-28

**Authors:** Sha Li, Chien-Shan Cheng, Cheng Zhang, Guo-Yi Tang, Hor-Yue Tan, Hai-Yong Chen, Ning Wang, Agnes Yuen-Kwan Lai, Yibin Feng

**Affiliations:** ^1^ School of Chinese Medicine, Li Ka Shing Faculty of Medicine, The University of Hong Kong, Hong Kong, China; ^2^ School of Public Health, Shanghai Jiao Tong University School of Medicine, Shanghai, China; ^3^ School of Nursing, The University of Hong Kong, Hong Kong, China

**Keywords:** COVID-19, virus infection, cytokine storm, immune response, organ protection, dietary supplements, functional foods

## Abstract

**Background:** The outbreak of the pandemic coronavirus disease 2019 (COVID-19) has now become a global pandemic spreading throughout the world. Unfortunately, due to the high infectiousness of the novel *β*-coronavirus, it is very likely to become an ordinary epidemic. The development of dietary supplements and functional foods might provide a strategy for the prevention and management of COVID-19.

**Scope and Approach:** A great diversity of potential edible and medicinal plants and/or natural compounds showed potential benefits in managing SARS, which may also combat COVID-19. Moreover, many plants and compounds have currently been proposed to be protective against COVID-19. This information is based on data-driven approaches and computational chemical biology techniques. In this study, we review promising candidates of edible and medicinal plants for the prevention and management of COVID-19. We primarily focus on analyzing their underlying mechanisms. We aim to identify dietary supplements and functional foods that assist in managing this epidemic.

**Key findings and Conclusion:** We infer that acetoside, glyasperin, isorhamnetin, and several flavonoid compounds may prevent and/or be effective in managing COVID-19 by targeting the viral infection, reducing the host cytokine storm, regulating the immune response, and providing organ protection. These bioactive dietary components (used either alone or in combination) might assist in the development of dietary supplements or functional foods for managing COVID-19.

## Introduction

The rapid global spread of coronavirus disease 2019 (COVID-19), which is caused by the novel *β*-coronavirus SARS-COV-2, poses significant threats to public health. The number of confirmed cases and deaths continues to grow worldwide. COVID-19 can develop rapidly into acute respiratory distress syndrome, resulting in multiple organ dysfunction or death in some cases (Singhal, 2020). Medical resources and experience in treatment are far from sufficient for conquering the virus. Similar to outbreaks of other newly identified viruses, COVID-19 patients are predominately managed with symptomatic therapies such as antiviral drugs (including lopinavir/ritonavir), often resulting in an unsatisfactory outcome (Ortiz-Prado et al., 2020). More importantly, due to the high infectiousness of SARS-COV-2, COVID-19 is very likely to become an ordinary epidemic that exists chronically. Therefore, dietary supplements or functional foods to prevent and manage viral infections might be of great importance.

**FIGURE 1:**
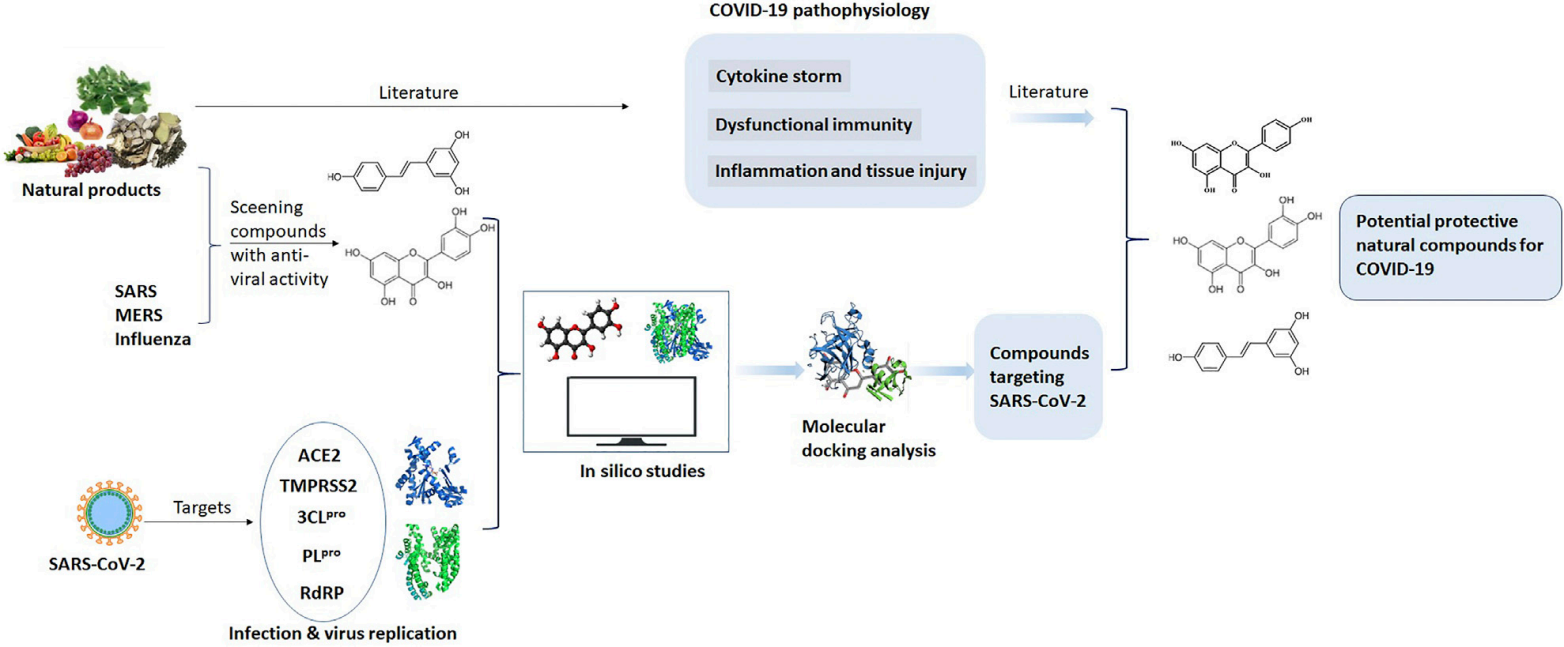
The scheme of this review.

Since the outbreak of SARS (caused by SARS-CoV) in 2003, many plants (including herbal tea and natural compounds) have been assessed for the prevention and treatment of *β*-coronavirus-associated diseases. Due to the homology in epidemiology, genomic, and pathogenesis of the SARS-CoV and SARS-CoV-2, the effect of edible and medicinal plants on SARS may also assist with managing COVID-19. Increasing evidence indicates that there are many similarities in the pathophysiological processes of the viral infections (SARS-CoV and SARS-CoV-2) in addition to direct lung injury, cytokine storm, dysfunctional immunity, as well as other organ injuries. Benefitting from the current understanding of COVID-19, various data-driven approaches and computational chemical biology techniques (such as molecular docking) have been adopted to screen potential natural compounds for managing COVID-19 (Ren et al., 2020). Within a short period, several large-scale screenings have been performed. A variety of promising natural herbal medicines and dietary bioactive compounds has been identified. In this study, we summarize the edible and medicinal plants that are potential COVID-19 management candidates. Figure 1 shows the framework of this review. We review the anti-inflammatory and immune-regulatory effects of these dietary bioactive compounds targeting SARS-CoV-2. The aim of this review is to provide the preliminarily research to uncover their molecular mechanism for managing COVID-19. This review provides important insights into the development of dietary supplements and functional foods from natural products for the prevention and management of COVID-19.

## Current Understanding of Pathophysiology of COVID-19

SARS-CoV-2 is a spherical, enveloped, positive-sense, single-stranded RNA coronavirus that shares approximately 80% similarity with the SARS-CoV genome (Machhi et al., 2020). It has four structural proteins, including spike glycoprotein, membrane glycoprotein, envelope protein, and nucleocapsid protein. The initial infection involves an interaction with a potential host cell. The spike proteins in SARS-CoV-2 are primed by the cellular transmembrane protease serine 2 (TMPRSS2) into S1 and S2 subunits. The S1 subunit specifically binds to the host cell receptors angiotensin-converting enzyme 2 (ACE2) or CD147 for entry, leading to a conformational change in the S2 subunit (Ni et al., 2020). ACE2 is also an entry receptor for SARS-CoV, whereas CD147 is a novel route for SARS-CoV-2 invasion. Functional S2 allows the infusion of viral and cellular membranes, allowing viral RNA to be released into the cytoplasm. Then, the viral genomic RNA begins to express copies of the virus in the host cell. The coronavirus replication involves papain-like protease (PL^pro^) and 3C-like protease (3CL^pro^), that hydrolyses the viral polyproteins pp1a and pp1ab to generate functional proteins (He et al., 2020). Afterward, the host cell transports copies of the virus to the cell surface, allowing the virus to infect other cells.

After SARS-CoV-2 infection, a well-coordinated and rapid innate immune response is activated (Vabret et al., 2020). The pathogen-associated molecular pattern (PAMP) of the virus is recognized by the pattern recognition receptor (PRR) on the membrane of the host cells, activating innate immune cells (such as macrophages, dendritic cells, monocytes, and neutrophils) to initiate the synthesis and secretion of inflammatory cytokines (Vabret et al., 2020). During viral infections, IL-6 and IL-1β can facilitate inflammation in the alveoli and bronchi. These are considered the major pro-inflammatory cytokines coordinating the local or systemic inflammation in infected individuals. Furthermore, cytokine storms might contribute to the impaired immune system. Ultimately, uncontrolled inflammatory responses cause the cytokine storm, leading to acute lung injury such as severe respiratory failure.

**FIGURE 2:**
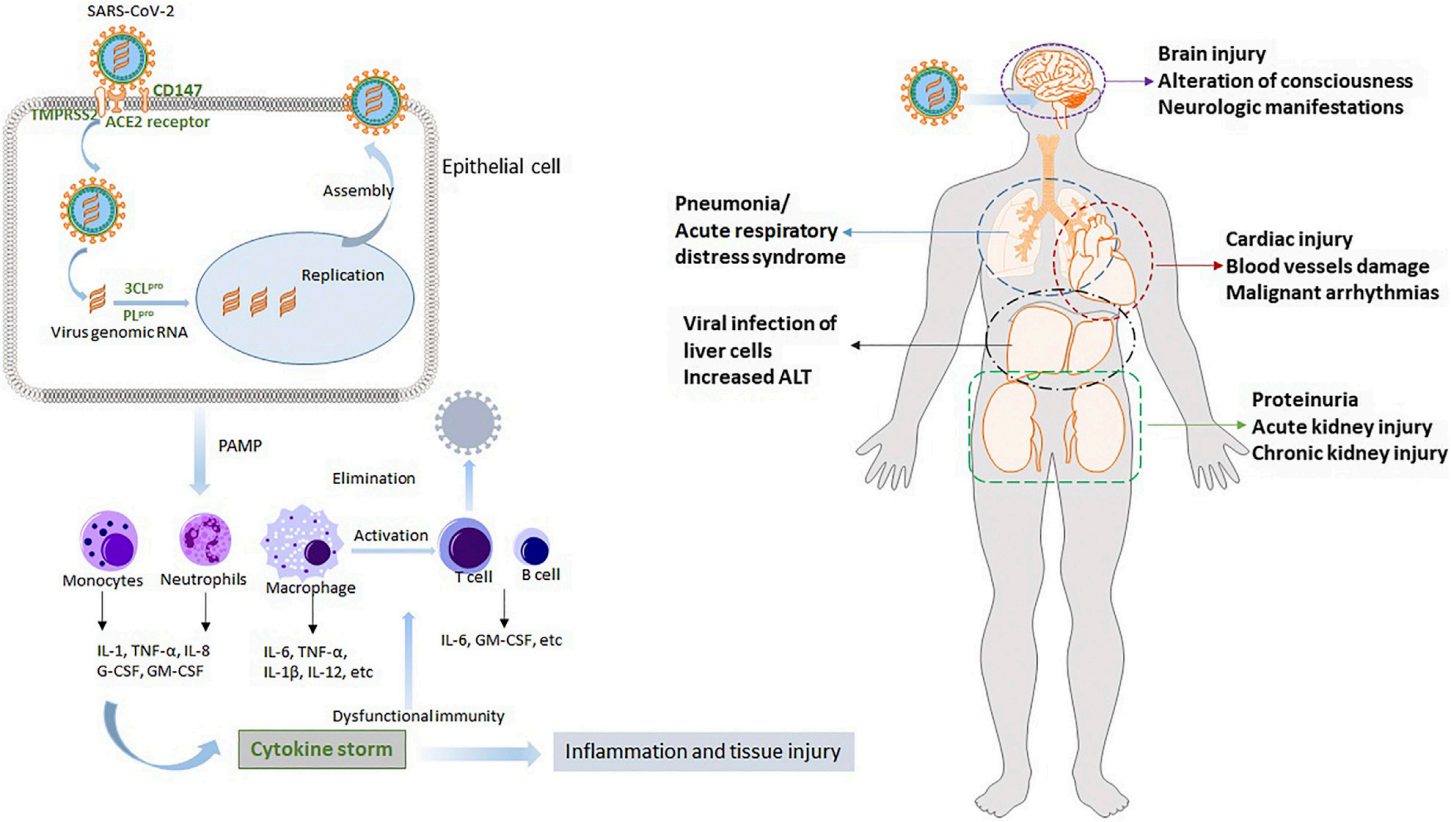
The potential pathogenesis of COVID-19 and its pathophysiology. After SARS-CoV-2 infection, rapid innate immune response is activated. Then uncontrolled inflammatory responses cause cytokines storm, leading to acute lung injury such as severe respiratory failure. Other multiple organs, such as heart, liver and kidney, are also injured due to the virus and cytokines storm.

In addition to lung injury, COVID-19 is regarded as a systemic disease involving multiple organs, such as the heart, liver, and kidney (Song et al., 2020; Wu et al., 2020; Zaim et al., 2020). After the initial infection in the respiratory system, SARS-CoV-2 disperses to other vital organs and tissues. This triggers a complicated spectrum of pathophysiological changes and symptoms. Multiple organ dysfunction is partially due to the wide expression of the cellular receptor ACE2 in these organs, and more importantly, results from the cytokine storm. Understanding the potential pathogenesis and pathophysiology of COVID-19 is indispensable for developing effective therapies and protective functional foods. We concisely review the SARS-CoV-2 infection process and the mechanisms underlying cytokine storm and organ injury. The results are provided in Figure 2.

## Potential Plants and Compounds

Many herbal plants, plant preparations, and phytoconstituents have a long history in antiviral therapy and play a vital role in preventing SARS transmission (Ho et al., 2020; Li et al., 2020b). Currently, clinical treatments for COVID-19 patients principally are symptomatic treatments such as anti-viral drugs and ventilator. However, since the physiopathologic processes involved in COVID-19 are complicated, therapies targeting on virus, systematic inflammation, immune response and organ protection together would benefit more for patients. Herbal plants and plant compounds, which are low toxic, cheap and easily available, are considered to treat both principal and secondary aspect of disease in Traditional Chinese Medicine. Many herbal plants are multi-target and multi-component pattern, which are promising for the prevention and management of COVID-19 in the future. The homology in the epidemiology, genomic, and pathogenesis of SARS-CoV and SARS-CoV-2 suggests that studies into effective edible and medicinal plants for SARS might also assist in managing COVID-19. Due to the low toxicity and availability of many natural products, screening plants or active compounds targeting SARS-CoV-2 or the host targets could be a potential strategy for managing COVID-19. In this section, we summarize the frequently used plants and derived natural compounds containing strong binding affinities with COVID-19 related targets.

### Edible and Herbal Plants

We analyzed the commonly used herbal formulae proposed for COVID-19. The herbal formulae contain 54 herbs, of which *Radix astragali* praeparata cum melle and *Glycyrrhizae Radix Et Rhizoma* are most frequently used. Other herbal plants of *Saposhnikovia divaricata (Turcz. ex Ledeb.) Schischk*, *Atractylodes macrocephala Koidz.*, *Lonicera japonica Thunb.*, *Forsythia suspensa* (Thunb.) Vahl, *Atractylodes lancea* (Thunb.) DC*.*, *Platycodon grandiflorus* (Jacq.) A. DC*.*, *Pogostemon cablin* (Blanco) Benth*.*, and *Cyrtomium fortune* J. Sm. are also often used (Luo et al., 2020). In a previous screening study based on data mining, molecular docking, and network pharmacology, 574 herbal prescriptions used for pestilence were obtained from 96,606 classical prescriptions (Ren et al., 2020). Among them, those high-frequency herbal plants were screened, including *Glycyrrhizae Radix Et Rhizoma, Scutellaria baicalensis Georgi, Rhei Radix Et Rhizome*, *Paeonia lactiflora Pall.*, *Citri Reticulatae Pericarpium*, *Bupleurum falcatum* L*.*, *Platycodon grandiflorus* (Jacq.) A. DC, *Atractylodes lancea* (Thunb.) DC, *Angelica sinensis* (Oliv.) Diels*,* and *Rehmannia glutinosa* (Gaertn.) DC*.* Notably, *Glycyrrhizae Radix Et Rhizoma*, *Platycodon grandiflorus* (Jacq.) A. DC and *Atractylodes lancea* (Thunb.) DC are regarded as high-frequency herbal plants in these two studies. Particularly, most of those commonly used herbal plants belong to dietetic herbs, such as *Glycyrrhizae Radix Et Rhizoma* (Liquorice)*, Lonicera japonica* Thunb*., Citri Reticulatae Pericarpium, Platycodon grandiflorus* (Jacq.) A. DC*.* and *Angelica sinensis* (Oliv.) Diels*,* which can be used as foods or sold as herbal tea.

A variety of vegetables and herbal plants has been tested against SARS-CoV. The extract of the tender leaf of the vegetable named Chinese mahogany (*Toona sinensis* (Juss.) M. Roem*.*), red spider lily (*Lycoris radiata* (L'Hér.) Herb*.*), and an extract of *Rhizoma Cibotii*, all inhibited SARS-CoV replication in vero cells with the SARS-CoV strain infection model (Panyod et al., 2020). Lianhua Qingwen capsule has been proven to be effective in Influenza A, Influenza B, Avian influenza. It has been selected as a general prescription for the treatment of COVID-19 in different stages that was later promoted to be used nationwide.

### Compounds

Due to the limited accessibility of SARS-CoV-2, a diverse array of studies adopted virtual simulation technologies (such as network pharmacology and molecular docking) to predict the potential bioactive component responses from natural products and the possible action mechanisms. These studies demonstrate some common ingredients and action mechanisms used by the edible and medicinal plants in treating SARS-CoV-2 infection.

A total of 166 herbal prescriptions containing 179 medicinal plants that have been proposed for use in treating COVID-19 were analyzed by network pharmacology. *β-Sitosterol*, stigmasterol, and quercetin were screened as the most frequently used compounds that are likely to be related to the antiviral signaling pathway. In another study using a screening system based on data mining, molecular docking, and network pharmacology, 431 chemicals from 35 high-frequency medicinal plants used for pestilence were molecularly docked with the SARS-COV-2 targets, ACE2 and 3CL^pro^, using LigandFit (Ren et al., 2020). A total of 48 compounds were docked with ACE2 and 27 compounds were docked with 3CL^pro^. The compounds were present in many edible and medicinal plants such as *Glycyrrhizae Radix Et Rhizoma*, *Scutellaria baicalensis* Georgi, *Rhei Radix Et Rhizome*, and *Bupleurum falcatum* L. Notably, acetoside (present in osmanthus flowers) showed the strongest binding activity to 3CL^pro^ (Consensus scoring = 7). In another dietetic herb (*Glycyrrhizae Radix Et Rhizoma*), glyasperin had the strongest binding activity to ACE site 1 (Consensus scoring = 6). Isorhamnetin showed the strongest binding ability to ACE site 2 (Consensus scoring = 6). Furthermore, as shown by the constructed compound-target network, quercetin, kaempferol, and baicalein (which are widely distributed in many vegetables, fruits, and medicinal plants) present high interconnection degrees, implying that these compounds regulate multiple disease targets (Ren et al., 2020). Emodin (an anthraquinone compound contained in various plants and several species of fungi) has been shown to suppress the binding of SARS-CoV S protein with ACE2 in a dose-dependent manner (Ho et al., 2007).

Many compounds belonging to flavonoids could target SARS-CoV-2 infection. Flavonoids are rich in many foods, including fruits, vegetables, and other plants. Production of inflammatory cytokines caused by the activation of the NLRP3 inflammasome in activated immune cells leads to respiratory distress syndrome which is associated with SARS coronaviruses (Chen et al., 2019). Various flavonoids have been shown to interfere with NLRP3 inflammasome signaling (such as wogonoside, baicalin, kaempferol, luteolin, myricetin, quercetin, and apigenin) and alleviate the inflammatory response to SARS-CoV infection (McKee et al., 2020). These compounds have also been demonstrated to be effective against various other viruses through multiple mechanisms. These compounds could be used as nutraceutical supplements at daily doses ranging from 100 to 500 mg. Resveratrol, a well-known natural polyphenol that is particularly abundant in grapes and sprouted peanuts, suppresses MERS-CoV infection and facilitates cellular survival after virus infection by inhibiting nucleocapsid protein (Lin et al., 2017). This suggests that these flavonoids might be promising health supplements or medical agents against SARS-CoV-2 infection.

The main constituent of *Nigella sativa*, thymoquinone, showed remarkable anti-oxidant, anti-inflammatory, anti-tumor, and antimicrobial activities (Seo et al., 2014; Ulasli et al., 2014; Ahmad., et al., 2020c). Notably, extract of *Nigella sativa* and thymoquinone have been demonstrated to be effective against avian influenza virus (H9N2 AIV) and in a murine cytomegalovirus infection model (Ahmad., et al., 2020c). Cells pre-treated with Nigella sativa extract reduced the replication of the virus when infected with coronavirus (Ahmad., et al., 2020c). Moreover, gene expression analysis of the transient receptor potential proteins (TRPs) indicated that Nigella sativa treatments decrease virus loads, thus reducing coronavirus survival inside cells. Thymoquinone has been demonstrated a remarkable anti-sepsis and immunomodulatory activities (Ahmad et al., 2013; Alkharfy., et al., 2015b; Raish et al., 2017; Alkharfy et al., 2018). It regulates the production of nitric oxide (NO) and reactive oxygen species (ROS), and prevented from multiple organ dysfunction syndrome (MODS) (Alkharfy., et al., 2015b; Raish et al., 2017; Alkharfy et al., 2018). Thymoquinone has been shown to protect against lung fibrosis and collagen deposition via regulating NF-κB and the antioxidant enzyme nuclear factor 2 heme oxygenase-1 (Nrf2/HO-1) signaling pathway (Ahmad., et al., 2020a).

## Possible Mechanisms Underlying the Action of Natural Products

The possible mechanisms underlying the action of natural products in treating COVID-19 mainly act on SARS-CoV-2, anti-inflammation, immunoregulation, and organ protection. We systematically review the potential action mechanisms of these natural products based on the available evidence. We particularly focus on the natural bioactive compounds that act on SARS-CoV-2 including acetoside, glyasperin, isorhamnetin, quercetin, kaempfero, baicalein, luteolin, and resveratrol (Table 1).

**TABLE 1:** The anti-inflammation and immune-regulation pathways mediated by compounds with strong ability to target on SARS-CoV-2 predicted by *in silico* studies (OB and Caco are used to evaluate the druggability of compounds; ↓ down-regulated or reduce; ↑ up-regulated or increase).

Compound	Representive plants or foods	Acts on SARA-CoV-2	Anti-inflammation		Immune-regulation		Bioavailability/OB/Caco	Refs
			Effects	Mechanisms	Effects	Mechanisms		
Isorhamnetin	*Glycyrrhizae Radix Et Rhizoma, Bupleuri Radix,* yellow onion, berry, grape	Binding with ACE2 and the main protease 3CL^pro^	Ameliorated LPS-induced inflammatory response	↓NF-κB signaling	Maintained immune regulation	JAK/STAT pathway	49.60/0.31	Sun et al., (2020b)
Quercetin	*Glycyrrhizae Radix Et Rhizoma etc.* grapes, berries, cherries, apples, citrus fruits, onions	Binding with ACE2 and the main protease 3CL^pro^	Reduced inflammation *in vivo* and *in vitro* induced by LPS and high-fat dies	↓TLR4/MyD88/PI3K; ↓NF-κB signaling	↑ Phenotypic expression of IFN-γ cells and decreased IL-4 positive cells	↓JAK/STAT pathway; ↓ SphK1/S1P signaling	46.43/0.05	Chen et al., (2016); Li et al., (2016)
Kaempferol	*Armeniacae Semen Amarum,* many fruits and vegetables such as grapes, apples, onions, spinach etc.	Binding ability to 3CL^pro^	Reduced the inflammation of LPS-treated macrophages and cardiac fibroblasts	↓ Src, Syk, IRAK1 and IRAK4 as well as activation of NF-κB and AP-1; ↓phosphorylation of PI3K and AKT	Reducing inflammatory cytokines in LPS-treated macrophages	Inactivation of NF-κB, AP-1, and JAK-STAT	41.88/0.26	Tran et al., (2009); Lee et al., (2018); Bian et al., (2019)
Baicalein	*Scutellaria baicalensis,* fresh onion	Inhibit 3CL^pro^	↓ TNF-α or IL-6 in mice with LPS-induced lethal endotoxemia	↓NF-κB or ERK1/2	↑CD4+Foxp3+ T cells and enhances intestinal barrier function	↓STAT3/4 in the JAK-STAT signaling pathway in T cells; ↓S1P-STAT3 signaling	33.25/0.63	Mabalirajan et al., (2013); Bae et al., (2016); Cheng et al., (2018); Wang et al., (2018); Xu et al., (2019); Xu et al., (2018)
6-gingerol	*Ginger (Zingiber officinale Roscoe)*	Binding with *P*L^pro^ in high affinity	↓Pro-inflammatory cytokines such as TNF-α, IL-1, and IL-8	↓I-κBα phosphorylation, NF-κB activation	Regulating the cell balance of Th17/Treg	↓FOXP3	35.64/0.54	(Tjendraputra et al., (2001); Verma et al., (2004)
Geniposide	*Gardenia jasminoides Ellis*	High docking score against TMPRSS2	↓LPS-Induced Mastitis in Mice	Regulating expression of TLR4, thus affecting the downstream NF-κB and MAPK signaling pathways	Geniposide could induce duct cell differentiation	JAK2/STAT3 pathway; ↓activation of SphK1 and S1P signal transduction	14.64/-1.7	Sun et al., (2020b); Song et al., (2014); Sun et al., (2020b); Yao et al., (2015)
β-sitosterol	*Isatis indigotica* root, avocados, pistachio nuts, pistachio nuts, almonds	Binding ability to 3CL^pro^	Decreasing inflammation on human aortic endothelial cells	Activation of multiple transcription factors	Reducing damage on macrophages	Inactivation of STAT1 and NF-κB is mediated by the activation of S1P	33.94/-0.44	Agrawal and Awad, (2011)
Stigmasterol	*Ophiopogon japonicas,* various vegetables, legumes, nuts, seeds	Involved in anti-viral pathway	↓Pro-inflammatory and matrix degradation mediators in osteoarthritis-induced cartilage degradation	Inhibition of the NF-κB pathway	Immune response in gastric cancer cells	Inactivate the JAK/STAT signaling pathway	43.83/1.44	Gabay et al., (2010); Li et al., (2018)
Acetoside	*Rehmanniae Radix,* verbena, lemon verbena, and olives	Strong binding activity to 3CL^pro^	Relieved LPS-induced acute lung injury	Inhibiting proinflammatory cytokine production and NF-κB activation	↓Inflammatory immune response in osteoarthritis rats	Via JAK/STAT signaling pathway	−/−	Jing et al., (2015); Qiao et al., (2019)
Resveratrol	Grapes and sprouted peanuts	ACE2	Reduced inflammation	↑sirtuin-1, ↓ NF-κB and ↓ activation of Nod-like receptor family pyrin domain containing-3 inflammasome	Enhanced antimicrobial defense	Via S1P signaling of cathelicidin antimicrobial peptide production through an NF-κB→C/EBPα-dependent mechanism	19.07/0.8	Park et al., (2013); Filardo et al., (2020)

### Direct Target on SARS-CoV-2

Edible and medicinal plants have unique antiviral advantages. They can affect the virus directly, impede its proliferation, and promote the secretion of IFN, this antiviral activity owes to their multi-component and multi-target pattern (Lin et al., 2014; Ma et al., 2015). Since ACE2 is the critical surface receptor initiating SARS-CoV-2 invasion into the host, excess soluble forms of ACE2 or ACE2 inhibitors could be a possible strategy to treat COVID 19 (Liu et al., 2020). A large number of compounds from edible and medicinal plants have been tested (by molecular docking) for binding with ACE2 (Table 1). Isorhamnetin, a compound which is highly concentrated in several vegetables (such as parsley and green bell peppers) shows a strong binding ability to ACE site 2 (Consensus scoring = 6) (Pandey et al., 2020). The two viral proteases, 3CL^pro^ and *P*L^pro^ are responsible for virus replication and packaging in host cells, which are also regarded as the key targets for SARS-CoV-2 infection (Pandey et al., 2020). Research involved the high-throughput molecular docking of 12,541 compounds from the TCMSP database with ACE2 and the main protease 3CL^pro^ (Gao et al., 2020). It was found that isorhamnetin and quercetin simultaneously show remarkable binding ability with these two proteins. Baicalin and its aglycon baicalein are flavonoids in many edible plants*,* which could inhibit 3CL^pro^ and demonstrate remarkable antiviral activity in cell-based systems. Several compounds from ginger, such as 8-gingerol, 10-gingerol, and 6-gingerol are found to bind with *p*L^pro^ in high affinity to inhibit SARS-CoV-2 replication (Dibakar et al., 2020).

Another protease regarded as a potential target due to an ability to block SARS-CoV-2 entry into host cells is TMPRSS2, which plays a critical role in the priming of viral spike proteins. Rahman et al. screened 30,927 natural compounds from a database of NPASS to mine potent inhibitors of TMPRSS2 (Rahman et al., 2020). After the initial physicochemical analysis, 2,140 compounds were recognized as potent candidates for further docking. A total of 85 compounds have binding energies comparable to or lower than the standard inhibitor camostat mesylate. Among them, geniposide (which is an important component of *Gardenia jasminoides* J. Ellis) showed the highest docking score against TMPRSS2 (Rahman et al., 2020).

### Anti-Inflammation

Clinical studies have indicated that the levels of IFN-γ, IL-6, TNF-α, IL-2, MCP-1, and other pro-inflammatory cytokines are significantly increased in severe or critical COVID-19 patients. Conversely, individuals infected with SARS-CoV-2 have increased levels of anti-inflammatory cytokines such as IL-10 and IL-4 (Coperchini et al., 2020; Hu et al., 2020). Given the vital role of cytokine storm in the pathogenesis of COVID-19, agents able to reduce the release of many cytokines involved in the initiation and progression of inflammation (such as IFN-γ, IL-6, and TNF-α) may assist in preventing the progression of the disease. Overwhelming evidence shows that many herbal plants, vegetables, fruits, and many natural compounds show remarkable anti-inflammatory abilities (Coperchini et al., 2020; Hu et al., 2020; Jafarzadeh et al., 2020).

The transcription factor NF-κB is a critical regulator in initiating and propagating optimal immune responses (Hayden et al., 2006). The constitutive activation of the NF-κB pathway is implicated in lung inflammatory immunopathology caused by respiratory viruses, including SARS-CoV (DeDiego et al., 2014). In a study of SARS-CoV-infected macaques (comparing adults with younger macaques) demonstrated increased NF-κB nuclear translocation and stronger host responses in aged macaques (DeDiego et al., 2014). Therefore, NF-κB/TNF-α is extensively targeted for the treatment of inflammation and increasing evidence indicates that many edible and medicinal plants used in COVID-19 alleviated inflammation via the NF-κB/TNF-α signaling pathway.

The major compounds targeting SARS-CoV-2 (such as isorhamnetin, acetoside, quercetin, kaempferol, baicalein, geniposid, gingerol and resveratrol) have demonstrated a remarkable ability in alleviating inflammation. Isorhamnetin is one of the active constituents in the medicinal plant *Hippophae rhamnoides* L*.,* as well as in parsley and green bell peppers and has been demonstrated to possess anti-oxidative stress and anti-inflammatory activities in many chronic inflammatory conditions. It ameliorates the LPS-induced inflammatory response via the inhibition of NF-κB signaling (Shi et al., 2018). Acetoside, which is abundant in many foods such as verbena, lemon verbena, and olives, can relieve LPS-induced acute lung injury by inhibiting proinflammatory cytokine production and NF-κB activation in both *in vitro* and *in vivo* studies (Jing et al., 2015). It also showed a positive effect in suppressing inflammation in osteoarthritic rats via the JAK/STAT signaling pathway (Qiao et al., 2019). Quercetin, is a polyphenol in many foods (such as grapes and onions) and has been shown to produce anti-inflammatory activity *in vitro* and in animal models by inhibiting NF-κB activation (Chen et al., 2016). Kaempferol is the major flavonoid aglycone that is widely distributed in various fruits and vegetables, including grapes, apples, raspberries, tomatoes, peaches, potatoes, broccoli, onions, brussel sprouts, lettuce, cucumbers, squash, green beans, and spinach etc. It produces anti-inflammatory activity *in vitro* and *in vivo* by several mechanisms. Kaempferol could reduce the inflammation of LPS-treated macrophages via direct suppression of Src, Syk, IRAK1, and IRAK4 as well as the activation of NF-κB and AP-1. It inhibits the LPS- and ATP-induced phosphorylation in PI3K and AKT in cardiac fibroblasts to reduce inflammatory injury (Tang et al., 2015). Kaempferol and some of its glycosides significantly decrease the release of NO and TNF-α in LPS-treated RAW 264.7 cells (Tran et al., 2009). Baicalein, a bioflavone component derived from the root of *Scutellaria baicalensis Georgi*, and is also rich in some varieties of onions, possesses various pharmacological properties used in treating many diseases (Cheng et al., 2018). Baicalein decreased the generation of TNF-α or IL-6 and inhibited the activation of NF-κB or ERK1/2 in mice with LPS-induced lethal endotoxemia. In mice with airway inflammation, baicalein can attenuate the symptoms through the inhibition of the NF-κB signaling pathway (Mabalirajan et al., 2013; Wang et al., 2018; Xu et al., 2019).

*β*-sitosterol, is one of the plant sterols and is also found in foods such as avocados, pistachio nuts and almonds, were effective in alleviating inflammatory reactions induced by immune responses (Paniagua-Perez et al., 2017). It has been demonstrated that the anti-inflammatory effect of *β*-sitosterol on human aortic endothelial cells may be mediated by the activation of multiple transcription factors. Stigmasterol, which is present in various vegetables, legumes, nuts, and seeds, inhibits several pro-inflammatory and matrix degradation mediators typically involved in osteoarthritis-induced cartilage degradation and partly through the inhibition of the NF-κB pathway (Gabay et al., 2010). Geniposide showed the highest docking score against TMPRSS2, it is widely used in Asia for the treatment of inflammatory diseases. Geniposide exerts anti-inflammatory effects by regulating the expression of TLR4, thus affecting the downstream NF-κB and MAPK signaling pathways (Song et al., 2014). Gingerol compounds from ginger bind to *P*L^pro^ with high affinity to inhibit SARS-CoV-2 replication; it also showed remarkable anti-inflammatory ability by reducing the synthesis of pro-inflammatory cytokines, such as TNF-α, IL-1 and IL-8 via the suppression of I-κBα phosphorylation and NF-κB activation (Tjendraputra et al., 2001; Verma et al., 2004; Mashhadi et al., 2013). Therefore, these compounds target SARS-CoV-2 directly or involved in anti-viral-related pathways with prominent anti-inflammatory abilities worthy of further research.

Additionally, an acid component of Ephedra polysaccharide (ESP-B4) alleviated pulmonary inflammation by reducing the generation of IL-6, TNF-α, IL-8, and MMP-9 (Liang et al., 2018). Bupleurum polysaccharides significantly protect the lungs from injury through the inhibition of P-selectin-mediated recruitment of neutrophils in an acute pneumonia model (Tong et al., 2014; Tong et al., 2018). Polysaccharides have been found to treat colitis by inhibiting NF-κB signaling and NLRP3 inflammasome activation (Cui et al., 2019).

### Immunoregulation

There is a vital role of the immune response in viral infections; an inappropriate and weak innate immune system response to viruses increases the release of inflammatory cytokines is considered the main factor encouraging COVID-19 (Catanzaro et al., 2020; Garcia, 2020; Tufan et al., 2020). After viral infection, the excessive inflammatory innate defense and impaired adaptive immune response may lead to tissue injury both at the site of viral entry and at the systemic level (Azkur et al., 2020; Li et al., 2020a). In populations with SARS-CoV-2 infection, the cytokine storm reflects widespread uncontrolled dysregulation of the host immune response. The altered immune signaling pathways or relevant molecular cascades triggered by SARS-CoV-2 infection may be developed as therapeutic targets or vaccines for COVID-19 patients (Azkur et al., 2020; Feng et al., 2020; Jamilloux et al., 2020).

#### Immune Cells

Changes in the innate and adaptive immune system in COVID-19 patients have been highlighted in several studies. A significant reduction in the absolute number of circulating CD4^+^ T cells, CD8^+^ T cells, NK cells, and B cells, as well as a decrease in monocytes, basophils, and eosinophils has been observed in patients with severe COVID-19 (Mazzoni et al., 2020; Sun et al., 2020a). In a retrospective clinical cohort study of 452 patients with COVID-19, a remarkably lower number of T helper cells and cytotoxic T cells exist in severe cases (Qin et al., 2020). Lymphocyte counts seem to be directly correlated with disease severity and mortality. The reason for lymphopenia might partially be attributed to the injured lymphatic organs expressing ACE2 receptors (Hajivalili et al., 2020; Lega et al., 2020).

The influence of some medicinal plants and natural compounds on immune cells has been investigated extensively previously. Herbal formulations can enhance enteric mucosal immune responses in mice with *Bacillus dysenteriae* and *Salmonella typhimurium* induced diarrhea by increasing the CD4^+^ and CD8^+^ T cell populations (He et al., 2007). In addition to herbal formulae, compounds such as baicalein and gingerol also regulate the immune cell population. In a mouse model of food allergies, baicalein induces CD4+Foxp3+ T cells and enhances intestinal barrier function (Grosso et al., 1989). One of the major components of ginger, 6-gingerol, showed efficacy in treating mice with dextran sulfate sodium (DSS)-induced colitis by regulating the cell balance of Th17/Treg cells (Sheng et al., 2020). Additionally, ginsenoside Rg3, a red-ginseng-derived compound, ameliorates acute exacerbation of chronic obstructive pulmonary disease (COPD) by suppressing neutrophil migration (Guan et al., 2020).

#### The IL-6/JAK/STAT Signaling Pathway

Signals transmitted by a large number of cytokines, lymphokines, and extracellular factors are transduced by JAK/STAT signaling to induce biological effects in cells such as hematopoietic and immune cells (Ghoreschi et al., 2009; Seif et al., 2017). IL-6 is one of the major activators of JAK/STAT signaling. The increased IL-6 in COVID-19 patients can bind to the glycoprotein (gp130) receptor and IL-6 receptor, thereby facilitating the downstream activation of JAK/STAT signaling (Catanzaro et al., 2020; Zhang et al., 2020). In turn, the activated JAK/STAT pathway further promotes the generation of IL-6 (Zhang et al., 2020). Drugs targeting IL-6/JAK/STAT signaling may assist in the treatment of COVID-19. In addition to monoclonal antibodies targeting IL-6, JAK inhibitors have been tested in several clinical trials on patients with COVID-19 (clinicaltrials.gov) (Atal and Fatima, 2020).

Recently, network pharmacology and computer-aided drug design in virtual screening studies, several dietary bioactive compounds have been identified as beneficial for COVID-19 treatment due to their regulation of the JAK-STAT signaling pathway, such as kaempferol, quercetin, and luteolin. Kaempferol significantly decreased the release of TNF-*α*, IL-6, IL-1*β*, VCAM-1 and ICAM-1 that had been induced by LPS. This result demonstrates Kaempferol’s negative mediation in TLR4, NF-κB, and STAT signaling in inflamed rat intestinal microvascular endothelial cells (Bian et al., 2019). In another study, kaempferol 7-O-*β*-D-glucoside decreased pro-inflammatory mediators through the inactivation of NF-κB, AP-1, and JAK-STAT in LPS-treated RAW 264.7 macrophages (Lee et al., 2018). In cholangiocarcinoma cells, the JAK/STAT pathway activated by the proinflammatory cytokines IL-6 and IFN-γ in CCA cells was inhibited by quercetin treatment. This result was demonstrated by a reduction in the up-regulated phosphorylated-STAT1 and STAT3 proteins in a dose-dependent manner (Senggunprai et al., 2014).

A variety of natural compounds from vegetables and fruits target SARS-CoV-2 using their anti-inflammatory abilities. They have also been shown to regulate the JAK-STAT signaling pathway in many laboratory studies. In a - DSS-induced colitis model, baicalein downregulated the mRNA expression of STAT3/4 in the JAK-STAT signaling pathway in T cells, facilitating its mediation of T cell proliferation (Xu et al., 2018). *β*-Sitosterol exerts anti-inflammatory effects on macrophages by suppressing STAT1 and NF-κB (Agrawal and Awad, 2011). Acetoside also showed a positive effect by suppressing inflammation in osteoarthritic rats via the JAK/STAT signaling pathway (Qiao et al., 2019). Stigmasterol has also been demonstrated to inactivate the JAK/STAT signaling pathway in gastric cancer cells (Li et al., 2018). Isorhamnetin principally maintained glucose homeostasis in myotubes by activating the JAK/STAT pathway (Jiang et al., 2019). Geniposide could induce duct cell differentiation via activation of the JAK2/STAT3 pathway in exocrine cells isolated from mouse pancreas (Yao et al., 2015). Resveratrol suppressed LPS-induced inflammation through the suppression of NF-κB and JAK/STAT signaling pathways (Filardo et al., 2020).

#### The Sphingosine-1-Phosphate Receptor One Pathway

Sphingosine-1-phosphate (S1P) is a crucial mediator of the immune response and plays a vital role in lymphocyte trafficking, vascular integrity, as well as the release of cytokines and chemokines (Proia and Hla, 2015; Catanzaro et al., 2020). S1P binds with G-protein coupled receptors one to five to mediate innate and adaptive immunity, including regulating the trafficking and migration of various types of immune cells. Notably, S1P receptor 1 (S1PR1) signaling can significantly attenuate the cytokines induced by influenza virus infection by targeting immune cells. This result suggests that S1P or S1PR1 signaling may be a potential target for COVID-19 treatment.

The anti-inflammatory effect of *β*-sitosterol on macrophages by the inactivation of STAT1 and NF-κB is mediated by the activation of S1P (Agrawal and Awad, 2011). Quercetin alleviated pulmonary fibrosis by inhibiting sphingosine kinase 1 (SphK1)/S1P signaling, as demonstrated by *in vivo* and *in vitro* studies (Zhang et al., 2018). In DSS-induced colitis mice, baicalein decreased the levels of inflammatory mediators and significantly downregulated the expression of SphK1, S1PR1, and p-STAT3 in the colon. This result implies that S1P-STAT3 signaling is involved in the mechanism underlying baicalein’s therapeutic effect on colitis (Yao et al., 2020). Geniposide also suppressed the activation of SphK1 and S1P signal transduction. It can significantly inhibit the level of S1P as well as the expression of S1PR1 and SphK1 in fibroblast-like synoviocytes (FLSs) (Sun et al., 2020b). Resveratrol enhanced antimicrobial defense via S1P signaling of cathelicidin antimicrobial peptide production by a NF-κB-C/EBPα-dependent mechanism. These compounds might mediate the immune response via S1P related signaling in COVID-19.

### Protecting Target Organs

Cardiovascular diseases in COVID-19 patients involves IL-6, ACE2, and angiotensin as the critical mediators that drive the pathological process. The increased IL-6 from activated macrophages and endothelial and smooth muscle cells after SARS-CoV-2 infection promotes the generation of MCP-1, upregulates the expression of cell adhesion molecules, and motivates the proliferation and migration of vascular smooth muscle cells, thus promoting atherogenesis (St Paul et al., 2020). Therefore, the level of circulating IL-6 may be a risk predictor of cardiovascular events in COVID-19. Furthermore, the destruction of lung tissue and the air-blood barrier allows the SARS-CoV-2 to continue to infect other organs via the ACE2 receptor (Zaim et al., 2020). The downregulation of ACE2 expression disrupts the balance between angiotensin I and II, converting them into angiotensin one to nine and one to seven, leading to the over-production of angiotensin II and organ damage. The increased angiotensin II interacts with the angiotensin II receptor type 1. This activates the JAK/STAT pathway to induce the generation of IL-6, forming a positive inflammatory feedback loop and ultimately causing vascular inflammation (Busse et al., 2020; de Abajo et al., 2020).

Natural products are particularly promising for organ protection from COVID-19 due to their multi-component and multi-target patterns. The inhibition of SARS-CoV-2 from binding to ACE2 as well as anti-oxidation, anti-inflammation, anti-apoptosis, and anti-fibrosis properties of natural products contribute to organ protection (Li et al., 2015; Lam et al., 2016). Several studies have indicated that some decoctions of herbal plants possess beneficial effects on many organs, implicating the regulation of TNF, MAPK, PI3K-Akt, Ras, and apoptosis signaling pathways (Chen et al., 2020). The active compounds of another herbal formula may act on ACE2, IL-6, and GM-CSF. Gancainin H and liquorice glycide E (from liquorice) act on ACE2, and glycyrin targets IL-6 and GM-CSF, orchestrating multiple signaling pathways to attenuate inflammation and viral infection, thus preventing lung and heart injury caused by SARS-CoV-2 (Shi-Ying et al., 2020).

## Conclusion and Prospect

**FIGURE 3:**
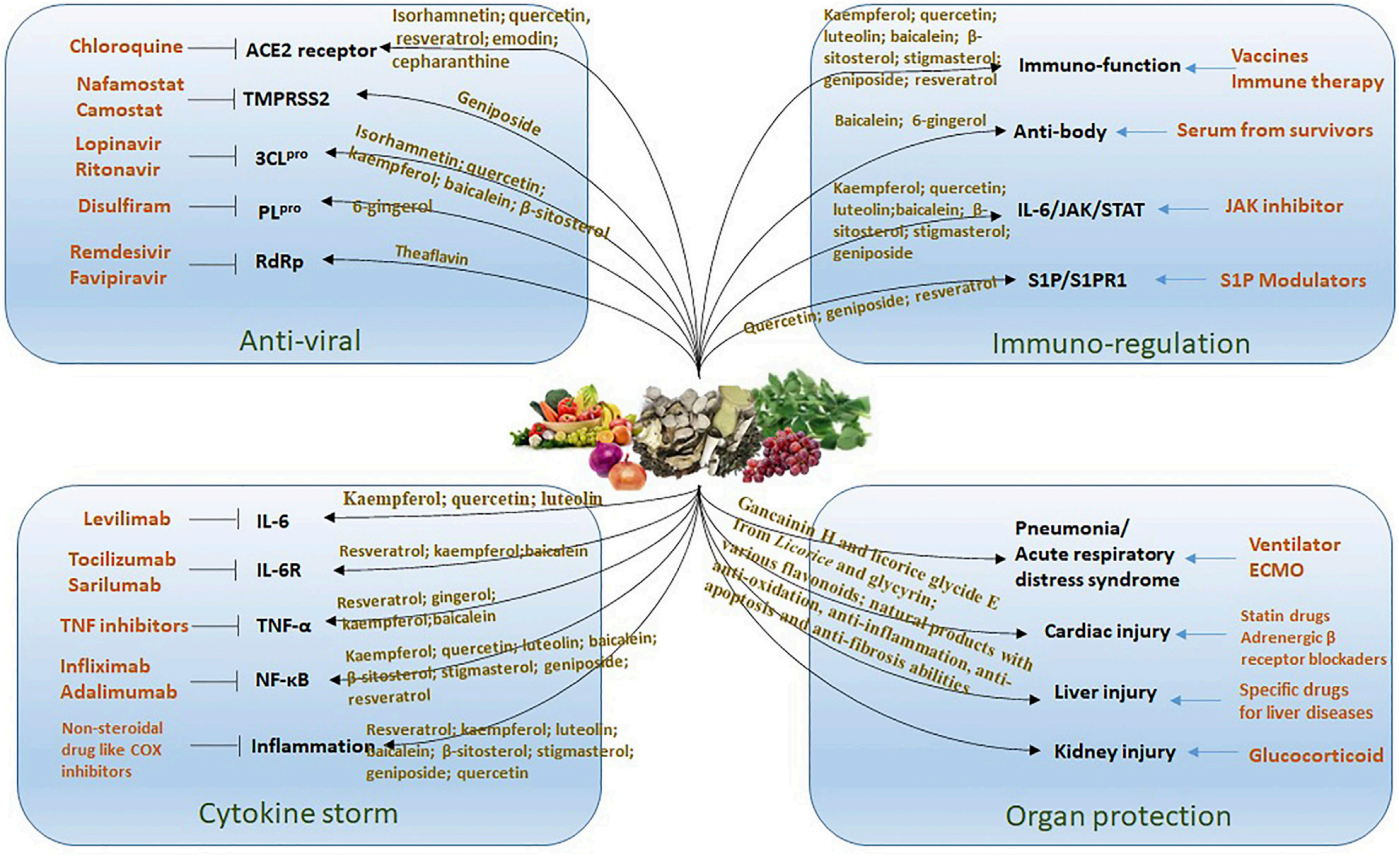
The treatment strategy for COVID-19 by using natural products. There are different drugs (list in orange color) acting on specific target for the treatment of COVID-19. The edible and medicinal plants have multiple compounds that targeting on multiple pathways to against virus infection including anti-viral, reducing cytokine storm, immuno-regulation and organ protection.

Presently, in managing the spreading epidemic, efforts should include normalizing the control of infection. Providing convincing and promising protective dietary components for people might be a strategy for preventing COVID-19. Evidence from available data, literature analysis, and *in silico* studies indicated that some bioactive compounds from edible and herbal plants are potentially protective against SARS-CoV-2 infection. These compounds are associated with antiviral, anti-inflammatory, immunoregulatory, and organ protection via cooperating multiple targets and pathways using various components (Figure 3). However, a limitation of the application of natural products in preventing and treating COVID-19 is due to our current understanding of the action mechanisms is mainly predictive using molecular docking and network pharmacology analysis. The active constituents, potential targets, and pathways predicted in these studies are not always consistent. Rigorous animal studies and trials on people are needed to verify these predictions. In this study, we reviewed the anti-inflammatory and immune-regulatory effects of the compounds predicted to possess a strong ability to target SARS-CoV-2 in experimental studies. Our research provides scientific evidence for their potency in the prevention and management of COVID-19. In conclusion, many dietary components with low toxicity and are easily available, such as flavonoids, acetoside, glyasperin, isorhamnetin, and ginger are promising candidates for the development of food supplements or functional foods for the prevention and management of COVID-19.
